# Comparison of nonparametric and parametric methods for time-frequency heart rate variability analysis in a rodent model of cardiovascular disease

**DOI:** 10.1371/journal.pone.0242147

**Published:** 2020-11-09

**Authors:** Emily M. Wong, Fern Tablin, Edward S. Schelegle

**Affiliations:** Department of Anatomy, Physiology and Cell Biology, School of Veterinary Medicine, University of California Davis, Davis, California, United States of America; Universidade Federal de Minas Gerais, BRAZIL

## Abstract

The aim of time-varying heart rate variability spectral analysis is to detect and quantify changes in the heart rate variability spectrum components during nonstationary events. Of the methods available, the nonparametric short-time Fourier Transform and parametric time-varying autoregressive modeling are the most commonly employed. The current study (1) compares short-time Fourier Transform and autoregressive modeling methods influence on heart rate variability spectral characteristics over time and during an experimental ozone exposure in mature adult spontaneously hypertensive rats, (2) evaluates the agreement between short-time Fourier Transform and autoregressive modeling method results, and (3) describes the advantages and disadvantages of each method. Although similar trends were detected during ozone exposure, statistical comparisons identified significant differences between short-time Fourier Transform and autoregressive modeling analysis results. Significant differences were observed between methods for LF power (*p* ≤ 0.014); HF power (*p ≤* 0.011); total power (*p* ≤ 0.027); and normalized HF power (*p* = 0.05). Furthermore, inconsistencies between exposure-related observations accentuated the lack of agreement between short-time Fourier Transform and autoregressive modeling overall. Thus, the short-time Fourier Transform and autoregressive modeling methods for time-varying heart rate variability analysis could not be considered interchangeable for evaluations with or without interventions that are known to affect cardio-autonomic activity.

## Introduction

Heart rate variability (HRV) is defined as the oscillation in the interval between consecutive heartbeats (i.e. RR interval), which is predominately influenced by the parasympathetic (PNS) and sympathetic (SNS) branches of the autonomic nervous system (ANS). Recognition of a significant association between the ANS and cardiovascular (CV) mortality has fueled use of HRV spectral analysis, a well-established, noninvasive technique that yields critical information on cardiac ANS activity, in clinical settings and research applications. In power spectral analysis, the frequency spectrum of an RR series is estimated, and subsequently, quantified into its main components; a typical spectrum is characterized by a low-frequency (LF) and high-frequency (HF) components. While the HF component is associated with respiration and modulated by PNS activity, the LF component is influenced by both ANS branches.

Importantly, ANS activity is dynamic; fluctuating over time as well as in response to various stimuli. To quantify changes in the HRV spectrum, several different time-varying or time-frequency approaches have been developed. Like traditional HRV spectral analysis, methods of time-frequency analysis fall into one of two categories, including; nonparametric, such as the short-time Fourier transform (STFT) [1,2], wavelet transform [3], and Wigner-Ville distribution [4,5]; and parametric, which are based on autoregressive (AR) model with time-varying coefficients [6]. While parametric methods are computationally more complex than nonparametric methods, they yield high resolution spectra that distinguishes each spectral component (i.e. LF and HF) independent of pre-selected frequency bands. Consequently, parametric methods provide a more accurate estimate of the power spectral density [7]. Although nonparametric and parametric methods are speculated to produce comparable results, previous studies have shown large discrepancies between traditional methods of HRV spectral analysis (i.e. fast Fourier transform and AR) [8–10]. However, nonparametric and parametric methods of traditional or time-frequency HRV spectral analysis have yet to be compared in rodents despite the ubiquitous use of HRV in rodent research models [11].

The objectives of the current study were to (1) compare how the nonparametric STFT and a parametric time-varying AR model influenced the time-frequency HRV spectra characteristics during an experimental exposure to O_3_ in mature adult spontaneously hypertensive (SH) rats, (2) evaluate the agreement and interchangeability between STFT and AR analysis methods, and (3) define the advantages and disadvantages of each analysis method.

In this study, we used SH rats because they are a well-characterized rodent model of cardiovascular disease (CVD) that demonstrate increased blood pressure, unprovoked atrial tachyarrhythmias and decreased HRV, comparable to human populations with CVD [12]. As a result, these animals provide a good model to compare STFT and AR analysis methods. An acute O_3_ exposure was chosen as a provocation because O_3_ is known to activate bronchopulmonary C-fibers [13] that in turn initiate cardiorespiratory reflexes that increase tonic PNS activity lowering heart rate [14]. In addition, O_3_-induced C-fiber activation results in the development of rapid shallow breathing [15] that was expected to reduce lung volume feedback from slowly adapting pulmonary stretch receptors, which is responsible for the development of respiratory sinus arrhythmia (RSA) and produces the distinct peak contained in the HF component of the HRV spectrum [16,17].

## Materials and methods

### Animals and housing

This study adheres to the ARRIVE guidelines for animal research [18]. The Institutional Animal Care and Use Committee at the University of California, Davis (Davis, CA) approved this study following guidelines mandated by the U.S. Federal Government [19]. Mature adult (45.7 wko [SD 6.9]; 367.1 g [SD 30.5]) male spontaneously hypertensive (SH, n = 11) rats were delivered from the vendors (Envigo Laboratories) and housed in filtered air in facilities approved by the American Association for Accreditation of Laboratory Animal Care. Rats were housed in a temperature- (22.0 ± 1.0°) and humidity-controlled (55.0 ± 5%) room with a 12 h light/dark cycle and air turnover 10 times per h.

#### Telemetry implantation

Rats were acclimated for at least five days in vivarium following delivery prior to undergoing surgical procedures. Rats were fasted for a minimum of 12 h prior to surgery. Prior to surgical procedures, rats were placed in a 10 L glass chamber and anesthesia was induced using 5% isoflurane and maintained through surgical procedures with 3–5% isoflurane (Abbott Laboratories, U.S.A.). A 0.1 to 0.5 ml blood sample was then obtained from a tail vein and analyzed for platelet and white blood cell counts. Following blood draw, rats were treated with meloxicam (2 mg/kg, SQ) and enrofloxacin (5 mg/kg, IM) and ophthalmic lubricant was applied to each eye. Abdominal, intercostal and femoral regions were shaved and skin surfaces were deeply scrubbed with a betadine solution. Following these injections and at least 10 min prior to surgery, incision sites were treated with multiple small subcutaneous injections of bupivacaine (5 mg/ml, total volume ≤ 0.25 ml). A 4–5 cm midline abdominal incision was made in the skin starting at the xiphoid and subcutaneous channels for the telemeter leads were formed using blunt dissection. A 2.0–2.5 cm incision was then made in the linea alba again starting at the xiphoid. A two-lead biopotential telemeter (TR50B Millar, Inc) was then placed in the abdominal cavity and secured to the lower abdominal wall using soluble suture. Incision in the linea alba was closed using monofilament suture and 10–15 cm of telemeter lead wire exited the closed incision near the xiphoid. Telemeter leads were secured using silk suture to the fascia covering the cranial portion of the sternum and the right eighth to tenth rib dorsal to the insertion of rectus abdominis. ECG signal quality was examined once telemeter leads were secured. These sites were chosen to minimize artifact associated with breathing and movement and produced a signal comparable to an aV2R ECG lead. Each surgical procedure lasted ~45 min. During recovery, rats were placed in a heated cage at ~37°C and were then individually housed in standard rat cages after their health condition was assured. During the recovery period, rats were monitored twice daily and analgesic therapies (see above) as well as buprenorphine (Buprenorphine (0.03 mg/kg), Par Phramaceutical) were administered twice daily for two days post-surgery.

### Experimental protocol

Rats were randomly assigned to one of two experimental exposure groups one week prior to study. On the day of study, rats were transferred into a standard rodent cage with an open wire mesh top with a perforated Teflon plate inserted ~1 inch above bottom of the standard cage. Rats were placed in exposure chamber at least 1 h prior to experimental exposure protocol. Rats underwent a whole-body exposure to either filtered air (FA; n = 6) or 1.0 ppm O_3_ (n = 7). Experimental exposure protocol was as follows: (1) 1 h FA baseline period (BL; 00:00:00 to 01:00:00); (2) 6 h exposure period (E1-E6; 01:00:00 to 07:00:00); (3) 8 h FA recovery period (R1-R8; 07:00:00 to 15:00:00). Rats had *ad libitum* access to water and chow for the entire duration of experimental protocol. Protocols began at ~5:00:00 pm (PST) and concluded the following morning at ~8:00:00 am (PST). A 12 h light/dark cycle was maintained during the protocols.

#### Ozone generation and monitoring

Ozone was generated, as described previously [15], by passing oxygen through an ozonizer (model 100, Sanders, Uetze-Eltze, Germany). After being mixed with filtered air, the gas was delivered (10 L/min) to the top compartment of the exposure chamber. All flows were controlled using mass flow controllers (Tylan General, San Diego, CA). The concentration of ozone was kept constant using a proportional controller (Inhalation Facility, University of California, Davis, California Regional Primate Research Center, Davis, CA) interfaced with an ultraviolet ozone analyzer (model 1003-AH, Dasibi Environmental, Glendale, CA). The ozone analyzer was calibrated using the ultraviolet absorption photometric method at the University of California, Davis, California Regional Primate Research Center.

#### Telemetry system and ECG recording

A telemetry-based biopotential amplifier and transmitter system (Telemetry Research Auckland, New Zealand) was utilized to measure ECG signals continuously throughout experimental exposure protocol. The unit incorporated an amplifier, 12-bit analog-to-digital converter sampling at 2 kHz, and transmitter (2.4-GHz band, range 5 m). The transmitter was encapsulated in medical-grade silicone and measured 35 × 23 × 11 mm, with a weight of 13 g. ECG signal from the transmitter was received via a dedicated receiver (Telemetry Research) that reconstructed the transmitted data signal. The reconstructed analog signal was acquired at a sampling rate of 4,000 Hz and displayed using the PowerLab data-acquisition system (model #ML870, AD Instruments, North America) and LabChart software (v8.0, AD Instruments, North America). This signal was band-pass filtered between 1 and 2,000 Hz.

### Time-frequency HRV analysis

ECG signal data were visually inspected to exclude artifacts including ectopic beats and arrhythmias; less than 3.8% (SD 1.2) of total beats were invalidated during these processes. R-wave time instances were extracted from the ECG recordings using an adaptive QRS detection algorithm to form RR interval time series data. HRV spectral analysis was performed using Kubios Heart Rate Variability (Premium v3.1 for Macintosh) software (Kubios, Kuopio, Finland) [20]. Prior to spectral analysis, RR series data were interpolated (5 Hz cubic spline interpolation) to produce evenly sampled data. In addition, ultra-low frequency trend components (<0.06 Hz) were removed using the smoothness priors method [21].

#### STFT analysis method

RR interval data series were analyzed using the short-time Fourier transform based on the fast Fourier transform [20]. Spectrum estimates were obtained using a 60-s moving window with 5-s shifts [22].

#### AR analysis method

RR interval series data were first modeled using a time-varying AR model, and then, model parameters were estimated recursively with the Kalman smoother algorithm [6]. A model order of 18 was selected based on model order selection criteria including Akaike’s final prediction error, Akaike information criterion, and minimum description length. The update coefficient of the Kalman smoother algorithm was set to 10^−4^ based on calculations using the error propagation formula as a good compromise between adaptation and estimate variance [6]. Spectrum estimates were obtained using a 60-s moving window with 5-s shifts.

We applied both the STFT and AR analysis methods to obtain the following HRV parameters for evaluation: Total spectral power (ms^2^), LF and HF component power (ms^2^) and peak frequencies (pHz; LF_p_ and HF_p_), as well as the LF/HF ratio. pHz data was obtained using AR method only. Absolute power for LF and HF components were calculated by integrating spectra over selected band limits: 0.2–0.8 Hz for LF and 0.8–2.5 Hz for HF. In addition, normalized units for HF power (HF_n_) were also calculated as, HF_n_ = [(HF power/(Total power–VLF power)) x 100%].

### Statistical analysis

The total number of rats was 5–6 per group (FA = 5; O_3_ = 6). All data are expressed as mean ± SEM. Data were analyzed using SPSS Statistics v25 (IBM, Armonk, NY). *p* Values ≤ 0.05 were considered statistically significant.

A one-way ANOVA with Bonferroni post-hoc comparison was used to identify significant differences between exposure groups for age (wko) and time (days) post-telemeter implantation (TI/E) on the day of experimental exposure.

Results from STFT and AR analyses were evaluated at the following time-points: baseline (BL; 00:55:00 to 01:00:00), exposure start (E0; 01:00:00–01:05:00), and exposure 1–11 (E1-11; 01:05:00–02:00:00). HRV parameter results represent the average of the 60-s spectrum data obtained within the five-min window specified for each time-point (S1 File).

Effects of Exposure, Time and analysis Method (STFT versus AR) were determined using a three-way mixed model analysis of variance (ANOVA) with Time and Method as within subject variables and Exposure as a between subjects variable, following procedures described previously by Littell *et al*. [23]. First, we determined the best fit of data to one of several within-subject covariance structures using the Schwartz Bayesian criterion and AIC; both of which indicated that the First Order Autoregressive: Heterogeneous covariance structure provided the best fit to data. Then, main effects of Exposure, Method and Time were analyzed by estimating and comparing means with a Bonferroni adjustment. Pearsons’ correlation coefficient was then used to compare STFT and AR analysis results.

We used the Bland-Altman method for determining agreement between methods of measurement with multiple observations to evaluate the interchangeability between STFT and AR analysis results overall and exposure-related observations [24]. First, a one-sample *t*-test was used to evaluate the differences between paired measure results from STFT and AR analyses. Differences between method results were then compared to their mean values using linear regression analysis (S2 File). Second, mean bias (*d*) and limits of agreement (LoA; lower and upper) were estimated using two different variances, one for the repeated differences between the two methods on the same subject and the other for the differences between the averages of the two methods across subjects. Within- and between subject variances were calculated using values obtained from a one-way ANOVA of the difference between paired method results. Total variance (TV) between methods across subjects was then calculated as the sum of the within and between subject variances. 95% confidence intervals (CIs) for mean bias (*d*) and LoAs were then estimated. For mean bias, CIs were calculated as [*d* ± (1.96 x *SD*_*TV*_)], where *SD*_*TV*_ is the standard deviation of the TV. 95% confidence intervals (CIs) were then calculated for the mean bias ([*d* ± (*t* x SE)]; where, SE = *SD*_*TV*_/√*n*), and the upper and lower LoA ([LoA ± (*t* x SE)], where SE = 1.71*s*_*d*_/√*n*). Acceptable LoA were calculated *a priori* as 150% of the mean difference between STFT and AR analysis result differences to represent parameter variability (Table 4).

In addition, a two-way repeated measures ANOVA with a Bonferroni adjustment was used to assess the effects of Exposure and Time on the RR interval (S3 File), as well as LF_p_ and HF_p_ obtained using the AR analysis method (S3 File).

## Results

### Animal characteristics on day of experimental exposure

Baseline animal characteristics are summarized in S1 Table. At the time of experimental exposure, there were no significant differences in age (wko; *p* = 0.977) or time post-telemeter implantation (TI/E; *p* = 0.651) between FA and O_3_-exposed rats.

### Time-related RR interval changes

**Fig 1:**
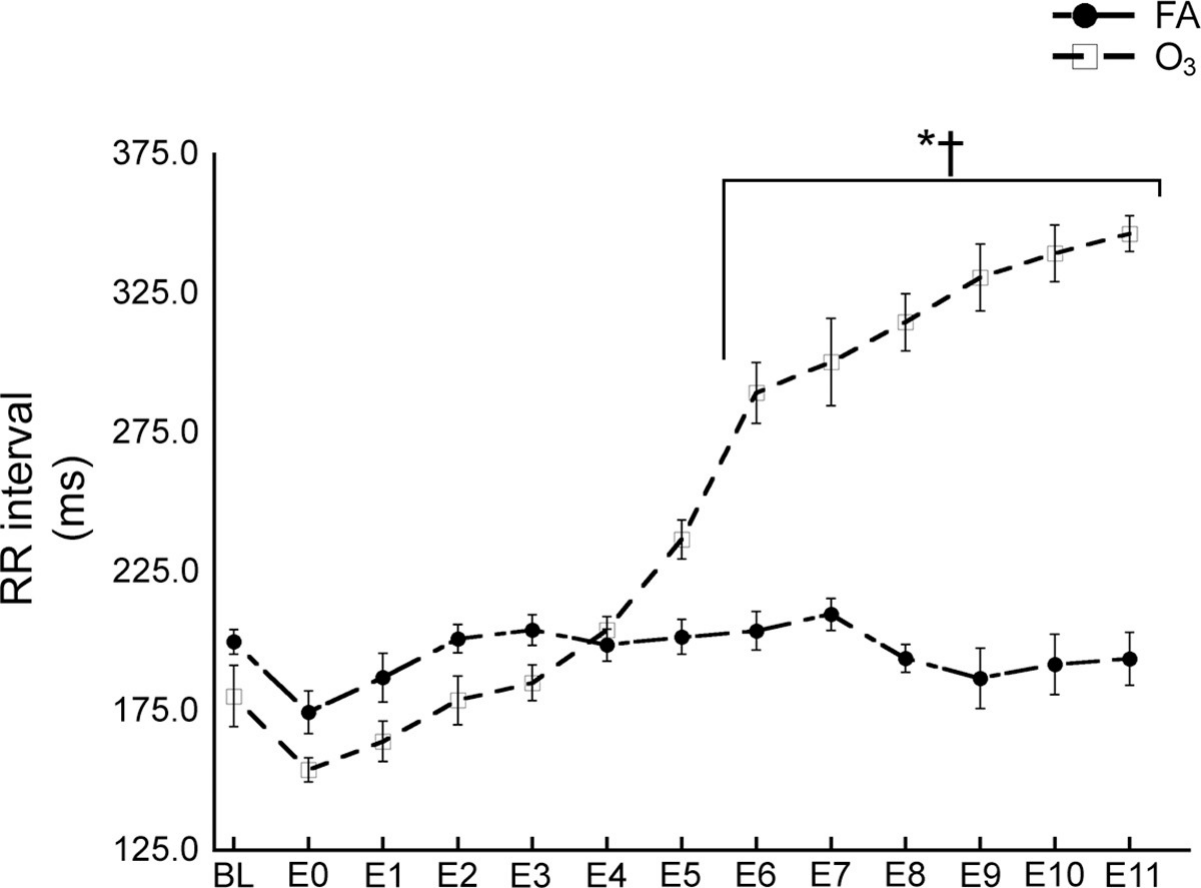
Time-related RR interval changes. O_3_ exposure increased the RR interval over time and compared to FA exposure. Abbreviations: O_3_, ozone; FA, filtered air; BL, baseline; E0, exposure start; E1-11, exposure time-points 1–11. Values are shown as the means ± SEM by exposure. *p* Values of ≤ 0.05 were considered statistically significant. *Significant difference between FA and O_3_. ^†^Significant difference from BL within O_3_ exposure group.

O_3_ significantly increased the RR interval compared to FA at E6-E11 (*p* ≤ 0.003). While FA-exposed rats showed no significant change in the RR interval over time, O_3_-exposed rats displayed increases from BL to E6-E11 (*p* ≤ 0.044) (Fig 1).

### Comparison of STFT and AR spectrum estimates

**Fig 2:**
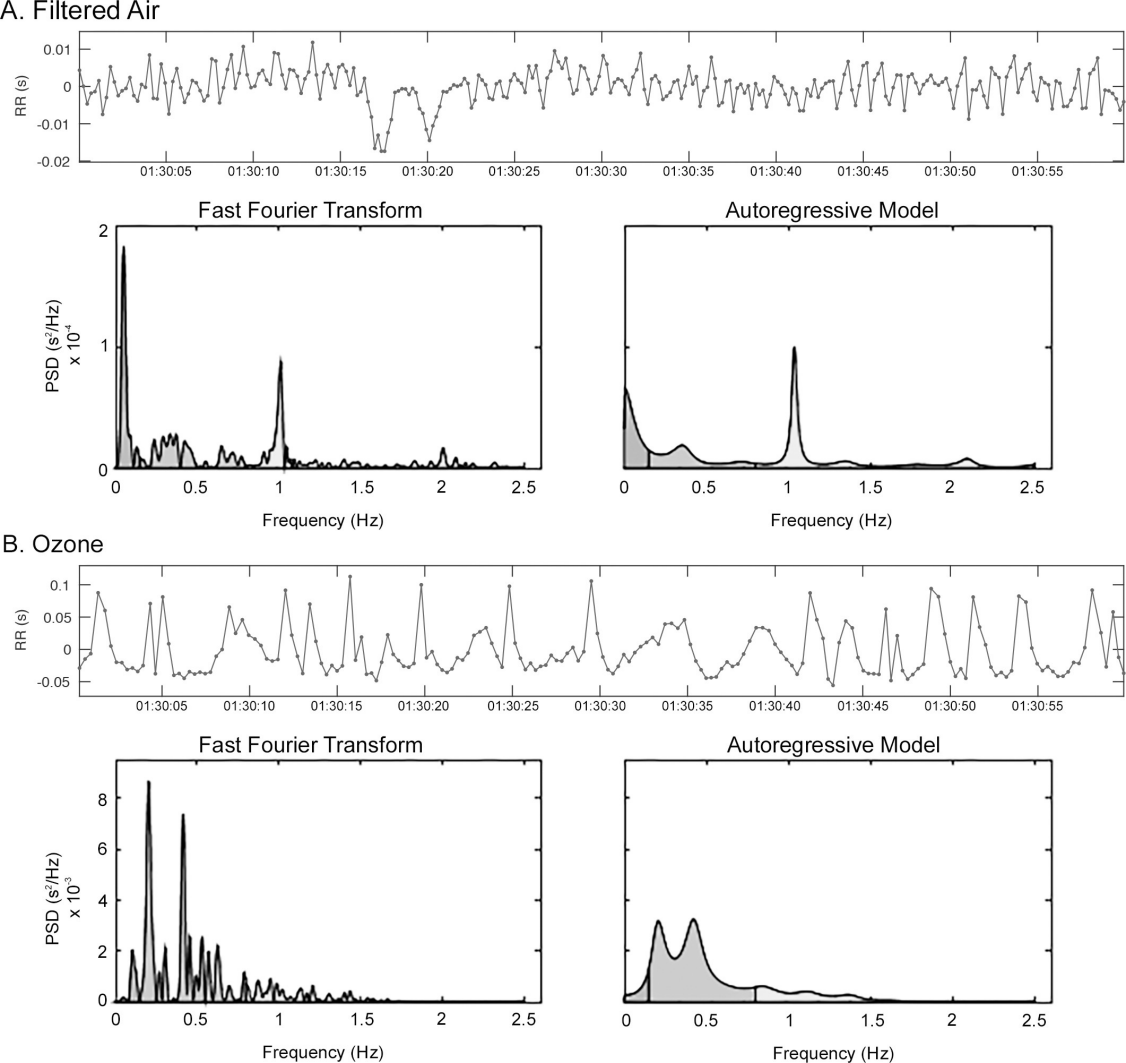
Representative tachogram of a one minute sample from a RR interval time series. (A) HRV spectral estimates from STFT and AR analysis methods for a FA-exposed rat and (B) HRV spectral estimates from STFT and AR analysis methods for a O_3_-exposed rat during E5 from 01:32:00 to 01:33:00.

Representative HRV spectrum estimates derived from STFT and AR analyses of a FA-exposed rat (Fig 2A) and a O_3_-exposed rat (Fig 2B) are presented in Fig 2. For FA, low resolution of the STFT spectrum estimate obscures key spectral characteristics (i.e. LF_p_) while improved resolution of the AR spectrum estimate clearly illustrates band power distribution and component pHz. For O_3_, the STFT spectrum estimate displays exposure-induced increases in spectral power and peak amplitude, however, multiple spectral peaks mask changes in component distribution. Alternatively, the AR spectrum estimate depicts exposure-induced shifts in LF and HF component distribution that are defined by a biphasic peak within the LF component and absence of a spectral peak in the HF component.

### Time-frequency HRV analysis results

**Fig 3:**
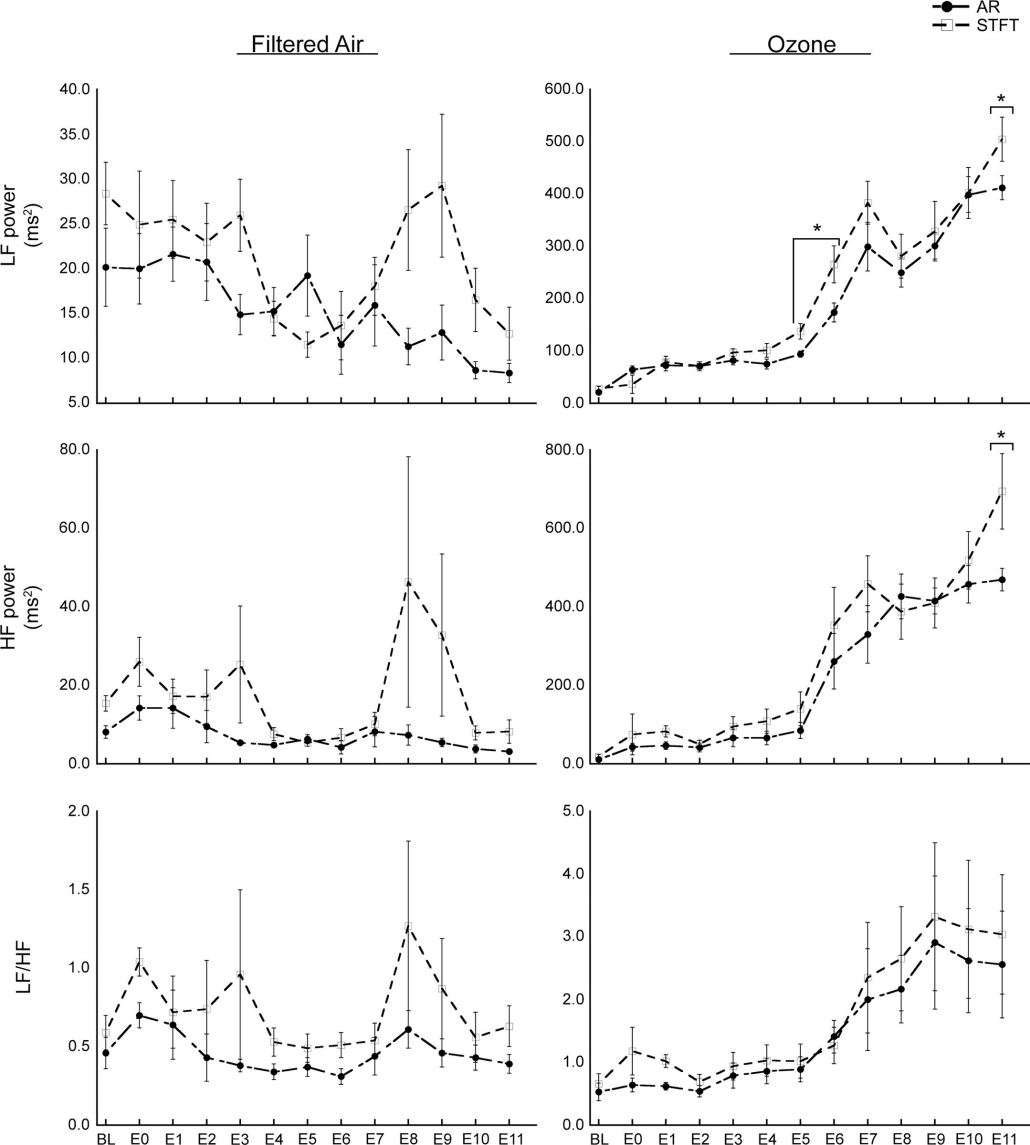
HRV parameter results from STFT and AR spectral analysis. Abbreviations: O_3_, ozone; FA, filtered air; BL, baseline; E0, exposure start; E1-11, exposure time-points 1–11. Results are shown as the means ± SEM by exposure group. *p* Values of ≤ 0.05 were considered statistically significant. *Significant difference between STFT and AR results.

STFT and AR spectral analysis results for LF power, HF power, and the LF/HF ratio are displayed in Fig 3; results for Total power and HF_n_ are displayed in S1 and S2 Figs.

#### STFT analysis

Isolating the results from STFT analysis, there were significant differences between FA and O_3_ exposure for Total, LF and HF powers at E1-E11; the LF/HF ratio at E9-E11; and HF_n_ at E3-E11. STFT analysis of FA-exposed rats showed no significant differences between time-points, while O_3_-exposed rats showed significant changes in LF power from BL to E2-E11; HF power from BL to E1 and BL to E3-E1; Total power from BL to E1-E11; and the LF/HF ratio from BL to E8-E11.

#### AR analysis

Isolating the results from AR analysis, there were significant differences between FA and O_3_ exposure for LF power at E0-E11; HF and Total powers at E1-E11; the LF/HF ratio at E6 and E8-E11; and HF_n_ at E4-E11. AR analysis of FA-exposed rats showed no significant difference between time-points, while O_3_-exposed rats, showed significant changes in LF power from BL to E0-E11; HF power from BL to E1 and from BL to E7-E11; Total power from BL to E1-E11; the LF/HF ratio from BL to E9-E10; and HF_n_ from BL to E8.

**Fig 4:**
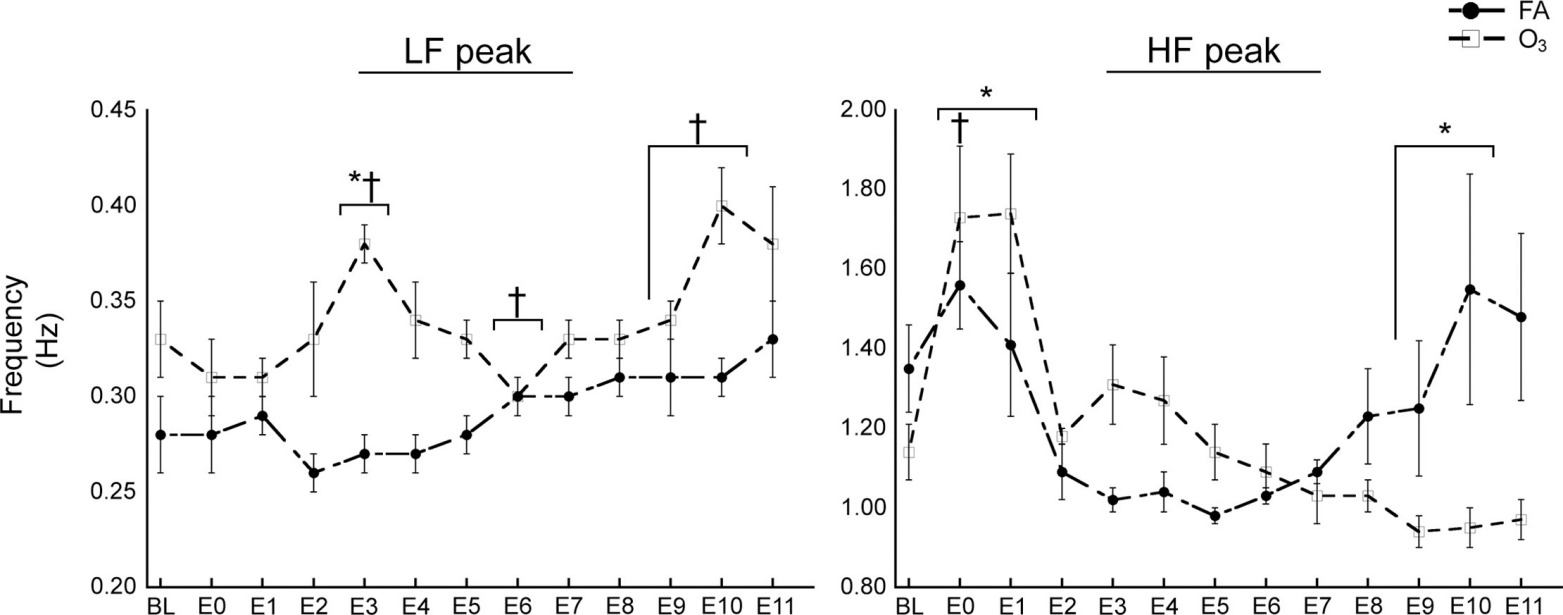
AR method detected O_3_-induced changes in HRV spectral component peak frequencies. (A) O_3_ altered LF_p_ and (B) HF_p_ over time. Abbreviations: O_3_, ozone; FA, filtered air; BL, baseline; E0, exposure start; E1-11, exposure time-points 1–11. Results are displayed as means ± SEM by exposure group. *p* Values of ≤ 0.05 were considered statistically significant. *Significant difference between FA and O_3_ exposure. ^†^Significant difference from BL within O_3_ exposure group.

In addition, we used the AR method to identify LF and HF component pHz. There were significant differences between FA and O_3_ exposure for LF_p_ at E3; and HF_p_ at E0-E1, E9-E10. FA-exposed rats showed no significant difference in LF_p_ or HF_p_ from BL to any other time-point, while O_3_-exposed rats, showed significant changes in LF_p_ from BL to E3, E6, E9-10; and HF_p_ from BL to E0 (Fig 4).

#### Comparison of STFT and AR method results

Results of the three-way mixed model ANOVA showed significant main effect of Method for LF power and HF_n_ (Table 1). Three-way interaction between Exposure, Time and Method were significant for all parameters except the LF/HF ratio and HF_n_ (Table 1). Isolating results for FA, there was a significant difference between STFT and AR results for HF_n_ at E11. Isolating results for O_3_, there were significant differences between STFT and AR results for LF power at E5-E6 and E11; HF power at E1 and E11; Total power at E5 and E11; and HF_n_ at E1. While STFT and AR results were significantly correlated for all parameters overall and with O_3_ exposure, HF power results were not correlated for FA exposure (Table 2).

**Table 1:** Main effects of three-way mixed model ANOVA. Abbreviations: LF, low frequency; HF, high frequency; LF/HF, the ratio between LF and HF components; HF_n_, normalized HF value. *p* Values of ≤ 0.05 were considered statistically significant.

Parameter	Exposure	Method	Time	Interaction
LF power	0.000*	0.022*	0.000*	0.000*
HF power	0.000*	0.067	0.000*	0.000*
Total power	0.000*	0.062	0.000*	0.000*
LF/HF	0.000*	0.207	0.002*	0.660
HF_n_	0.000*	0.033*	0.007*	0.283

*Significant main effect.

**Table 2:** Pearson’s correlation coefficient. Correlation between STFT and AR method results were determined using Pearson’s coefficient overall and within exposure-related groups.

Parameter	FA	O_3_	Overall
LF power	0.279	0.904*	0.938*
HF power	0.183	0.899*	0.927*
Total power	0.424	0.912*	0.945*
LF/HF	0.511*	0.963*	0.940*
HF_n_	0.691*	0.781*	0.810*

Abbreviations: FA, filtered air; O_3_, ozone; LF, low frequency; HF, high frequency; LF/HF, the ratio between LF and HF components; HF_n_, normalized high frequency power. *p* Values of ≤ 0.05 were considered statically significant.

*Significant correlation between STFT and AR method results.

#### Agreement between STFT and AR methods

STFT and AR method results for FA and O_3_ exposure groups were assessed for agreement both separately and overall. Overall, results of the Bland-Altman demonstrated large discrepancies between STFT and AR methods for all parameters; each exceeded accepted LoA set *a priori* (Table 4). Alternatively, linear regression analysis showed a significant correlation between method result differences and their mean value for all HRV parameters, except HF_n_ (Table 3). When data for the FA group alone was similarly compared, results from the Bland-Altman showed no agreement between STFT and AR methods for any parameter (Table 4). However, there were significant correlations between method result differences and their mean value for all parameters (Table 3).

**Table 3:** Linear regression of STFT and AR result differences compared to mean values. Linear regression was used to determine whether differences between STFT and AR results correlated with mean values.

Parameter	FA	O_3_	Overall
LF power	0.100*	0.161*	0.199*
HF power	0.809*	0.207*	0.251*
Total power	0.390*	0.241*	0.291*
LF/HF	0.580*	0.282*	0.215*
HF_n_	0.102*	0.000	0.000

Abbreviations: FA, filtered air; O_3_, ozone; LF, low frequency; HF, high frequency; LF/HF, the ratio between LF and HF components; HF_n_, normalized high frequency power. R^2^ values are displayed. *p* Values of ≤ 0.05 were considered significant.

*Significant correlation between method result differences and their mean value.

**Table 4:** Agreement between STFT and AR methods for time-frequency HRV analysis. Agreement between methods was assessed using the Bland-Altman method for multiple observations. Accepted LoA were determined *a priori* as 150% of mean difference between method results. Mean bias and LoA, upper and lower, were calculated by exposure group and overall.

All Observations				
Parameter	Mean Bias	Lower LoA	Upper LoA	Accepted LoA
LF power	19.3 (-0.1 to 38.7)	-91.3 (-124.5 to -58.2)	129.9 (96.8 to 163.1)	± 28.9
HF power	32.9 (-0.2 to 66.1)	-138.4 (-195.0 to -81.7)	204.2 (147.6 to 260.9)	± 49.4
Total power	51.0 (-0.4 to 102.4)	-239.2 (-327.1 to -151.4)	341.2 (253.3 to 429.1)	± 76.5
LF/HF	0.3 (0.0 to 0.6)	-0.7 (-1.2 to -0.2)	1.3 (0.8 to 1.8)	± 0.4
HF_n_	4.0 (0.0 to 8.0)	-13.8 (-20.7 to -6.9)	21.8 (14.9 to 28.6)	± 6.0
FA Observations				
LF power	5.4 (0.6 to 10.1)	-16.9 (-25.0 to -8.8)	27.6 (19.5 to 35.8)	± 8.0
HF power	10.1 (0.9 to 19.3)	-41.1 (-56.9 to -25.3)	61.3 (45.5 to 77.1)	± 15.2
Total power	7.1 (0.8 to 13.4)	-33.9 (-44.6 to -23.1)	48.1 (37.4 to 58.8)	± 10.7
LF/HF	0.3 (0.0 to 0.5)	-0.8 (-1.2 to -0.3)	1.3 (0.9 to 1.7)	± 0.4
HF_n_	5.0 (0.5 to 9.5)	-13.1 (-20.9 to -5.4)	23.1 (15.4 to 30.8)	± 7.5
O_3_ Observations				
LF power	30.9 (0.7 to 61.2)	23.7 (-28.0 to 75.3)	38.2 (-13.5 to 89.9)	± 46.4
HF power	51.9 (2.3 to 101.6)*	43.9 (-40.9 to 128.8)	60.0 (-24.9 to 144.8)	± 77.9
Total power	87.5 (3.9 to 171.1)*	79.6 (-63.4 to 222.5)	95.5 (-47.5 to 238.4)	± 131.3
LF/HF	0.3 (0.0 to 0.6)	-9.4 (-9.9 to -8.9)	10.0 (9.5 to 10.5)	± 0.4
HF_n_	3.2 (0.1 to 6.3)	-3.1 (-8.4 to 2.1)	9.5 (4.2 to 14.8)	± 4.8

Abbreviations: FA, filtered air; O_3_, ozone; LF, low frequency; HF, high frequency; LF/HF, the ratio between LF and HF components; HF_n_, normalized high frequency power. *Values within accepted limits of agreement.

When data for the O_3_ group alone was similarly compared, results from the Bland-Altman showed agreement between STFT and AR method results for HF and Total power but no other parameter (Table 4). Finally, there were significant correlations between method result differences and their mean value for all parameters, except HF_n_ (Table 3).

## Discussion

The aim of time-frequency HRV spectral analysis is to quantify changes in the HRV spectrum during non-stationary events, and thus, offer a distinct advantage over traditional spectral analysis methods that assume signal stationarity. In the current study, we evaluated the interchangeability and agreement between the most common nonparametric and parametric methods for time-frequency HRV spectral analysis in a rodent model of CVD during an acute O_3_ exposure. Comparison of STFT and AR analyses showed a significant effect of analysis Method on LF power and HF_n_. While there were significant correlations between STFT and AR analysis results overall and with O_3_ exposure, FA exposure results for HF power were poorly correlated. Furthermore, Bland-Altman analysis results demonstrated that agreement between STFT and AR analyses was inconsistent, suggesting poor interchangeability.

Prior to HRV data analysis, pre-processing techniques are applied to RR interval time series to minimize corruption and increase quality. Here, we first applied a cubic spline interpolation to convert the RR interval data series into an equidistantly sampled time series [25]. Second, a threshold-based artifact correction algorithm was employed to identify and correct technical artifacts [20]. Technical artifacts typically result from errors in measurement of R-wave occurrence times or algorithm QRS complex detection. Physiologic artifact (i.e., ectopic beats and arrhythmic events) were visually identified and removed prior to derivation of RR interval data time series. As a final step, data were detrended using the smoothness priors method, which involves a time-varying high-pass filter with an adjustable cut-off frequency [21]. While data pre-processing is not the central focus of our investigation, each of these pre-conditioning steps influences analysis results. Therefore, it is critical to identify the pre-processing techniques employed.

In this study, we compared the nonparametric STFT and a parametric method based on a time-varying AR model. In the STFT method, a moving-window FFT is used to estimate the spectra within a specified window as a function of time. In the parametric method, a time-varying AR model is first used to model the non-stationary signal; then, model parameters are estimated recursively with the Kalman smoother algorithm. The resulting spectral characteristics of which are dependent on model order [6]. Here, a model order of 18 was selected as a good compromise for all subjects (i.e. FA- and O_3_-exposed). In both STFT and AR analyses, LF and HF component powers were obtained by integrating the spectrum over specific frequency bands, which were set to 0.2–0.8 Hz and 0.8–2.5 Hz, respectively. These band limits have been applied by several studies of HRV in rodents [26–29] but differ from limits used by previous environmental exposure studies in rodents [30–32].

Acute inhalation of O_3_ activates bronchopulmonary C-fibers [16], increasing tonic PNS output while decreasing slowly adapting receptor activity in the lung secondary to the development of rapid shallow breathing (22). We expected that O_3_-induced lung C-fiber activation would increase PNS activity in the heart, altering HRV spectral component characteristics to reflect change in ANS activity. Our analysis indicates that quantifying changes in spectral component power alone may not be sufficient to characterize non-stationary events.

Importantly, HRV spectrum properties (i.e. power, pHz, and distribution) are highly individual, and vary with chronic physiologic conditions (e.g., age, presence of disease, etc.) [7,33]. Therefore, it is critical to define all HRV spectral characteristics when evaluating non-stationary events. As stated previously, component powers were obtained by integrating the spectrum over specific frequency bands. Pre-selected HRV component bandwidths assume that spectral characteristics are the same between subjects, and thus, changes in component power (absolute and relative) account for inter-animal variability as well as changes over time. These assumptions appear to hold when examining the O_3_ data where STFT and AR analysis results were highly correlated. With O_3_ exposure, however, there was a clear shift in power to the LF component at E6 (Fig 2B). Using STFT analysis, this O_3_-induced shift could only be characterized by increases in LF power and the LF/HF ratio. Alternatively, AR analysis demonstrated the appearance of a biphasic peak in the LF component that coincided with the loss of a clear HF peak (Fig 2B). This biphasic peak in the LF component was present in the spectra of all O_3_-exposed rats at E6. While spurious peaks can result from too high-model order, lower model orders did not affect the appearance of biphasic peak in the LF component but failed to distinguish a HF component peak (data not shown). Further investigation is needed to define the biologic basis for this phenomenon.

The Bland-Altman method is widely accepted method for comparing biologic measures because it accounts for inter-individual variability, a critical factor in physiologic evaluations. Although two different variances; one for repeated differences between two methods on the same subject and the other for differences between the averages of the two methods across subjects, were incorporated into the Bland-Altman analysis, results exceeded accepted limits for all parameters overall; HF and Total power for FA; HF power and the LF/HF ratio for O_3_. Critically, the Bland-Altman assumes that the repeated differences for a single subject are independent, and as a result, considered a conservative method for comparing methods.

Poor correlation between STFT and AR analysis results for FA combined with consistent observations of significant differences for LF, HF, and Total power illustrate the influence of spectral resolution on analysis results. This effect may be of limited importance when obtaining data over shorter time-periods with an intervention. When examining HRV over a prolonged period without an intervention (i.e. FA exposure), however, changes in component pHz significantly contributed to the difference and increased the mean difference between STFT and AR analysis results for HF power. Thus, these component characteristics may provide critical information on how ANS input to the heart changes over time.

This is the first study, to our knowledge, comparing nonparametric and parametric methods for time-frequency HRV spectral analysis in rodents. Although similar trends were detected over time and with O_3_ exposure, statistical comparisons identified significant differences between STFT and AR analysis results for all HRV parameters. Furthermore, the Bland-Altman method for agreement identified several inconsistencies within exposure-related observations, accentuating the lack of agreement between STFT and AR analysis methods overall. Thus, STFT and AR methods for time-varying HRV analysis could not be considered interchangeable for evaluations with or without interventions that are known to affect ANS input to the heart over a prolonged time-period in rodents.

## Conclusions

Both STFT and AR methods for time-frequency HRV analysis present distinct advantages as well as disadvantages. The STFT method is the most simple and straightforward, however, the frequency resolution over time is poor and statistically unreliable. While computationally demanding, the AR method yields insight on additional spectral characteristics by resolving the central or peak frequency of each component. Here, we found that the improved resolution of the AR method provided unique insights into O_3_ effect on parasympathetic activity.

## Supporting information

S1 Fig STFT and AR spectral analysis results for total power. Abbreviations: O_3_, ozone; FA, filtered air; BL, baseline; E0, exposure start; E1-11, exposure time-points 1–11. Results are shown as the means ± SEM by exposure group. *p* Values of ≤ 0.05 were considered statistically significant. *Significant difference between STFT and AR results.
(TIF)

S2 Fig STFT and AR spectral analysis results for HF_n_. Abbreviations: O_3_, ozone; FA, filtered air; BL, baseline; E0, exposure start; E1-11, exposure time-points 1–11; HF_n_, normalized high frequency power. Results are shown as the means ± SEM by exposure group. *p* Values of ≤ 0.05 were considered statistically significant. *Significant difference between STFT and AR results.
(TIF)

S1 Table Baseline animal characteristics. Mean age, weight, and number of weeks post-telemeter implantation of rats on day of experimental exposure. Abbreviations: FA, filtered air; O_3_, ozone; wko, age in weeks; TI/E, time between telemeter implantation and experimental exposure. Values are shown as means ± SEM.
(DOCX)

S1 File STFT and AR analysis method results. Data file for three-way mixed model ANOVA. Column designations: Exposure– 1 = FA, 2 = O_3_; Method– 1 = STFT, 2 = AR; Time– 1 = BL, 2 = E0, 3–13 = E1-E11. Abbreviations: FA, filtered air; O_3_, ozone; LF, low frequency power; HF, high frequency power; LF/HF, the ratio between LF and HF power; HF_n_, normalized HF power.
(XLSX)

S2 File STFT and AR result differences and mean value. Data file for linear regression analysis. Column designations: Exposure– 1 = FA, 2 = O_3_; Time– 1 = BL, 2 = E0, 3–13 = E1-E11. Abbreviations: FA, filtered air; O_3_, ozone; LF, low frequency power; HF, high frequency power; LF/HF, the ratio between LF and HF power; HF_n_, normalized HF power; mean, STFT and AR result mean; difference, difference between STFT and AR results.
(XLSX)

S3 File RR interval, LF_p_ and HF_p_. Data file for two-way repeated measures ANOVA of RR interval, LF_p_ and HF_p_. Column designations: Exposure– 1 = FA, 2 = O_3_; Time– 1 = BL, 2 = E0, 3–13 = E1-E11. Abbreviations: FA, filtered air; O_3_, ozone; LF_p_, low frequency component peak; HF_p_, high frequency component peak.
(XLSX)
