# Supplementary figures and images for: Comparison of nonparametric and parametric methods for time-frequency heart rate variability analysis in a rodent model of cardiovascular disease
Source: PLoS One. 2020 Nov 9;15(11):e0242147. doi: 10.1371/journal.pone.0242147 (PMC7652293; doi:10.1371/journal.pone.0242147)

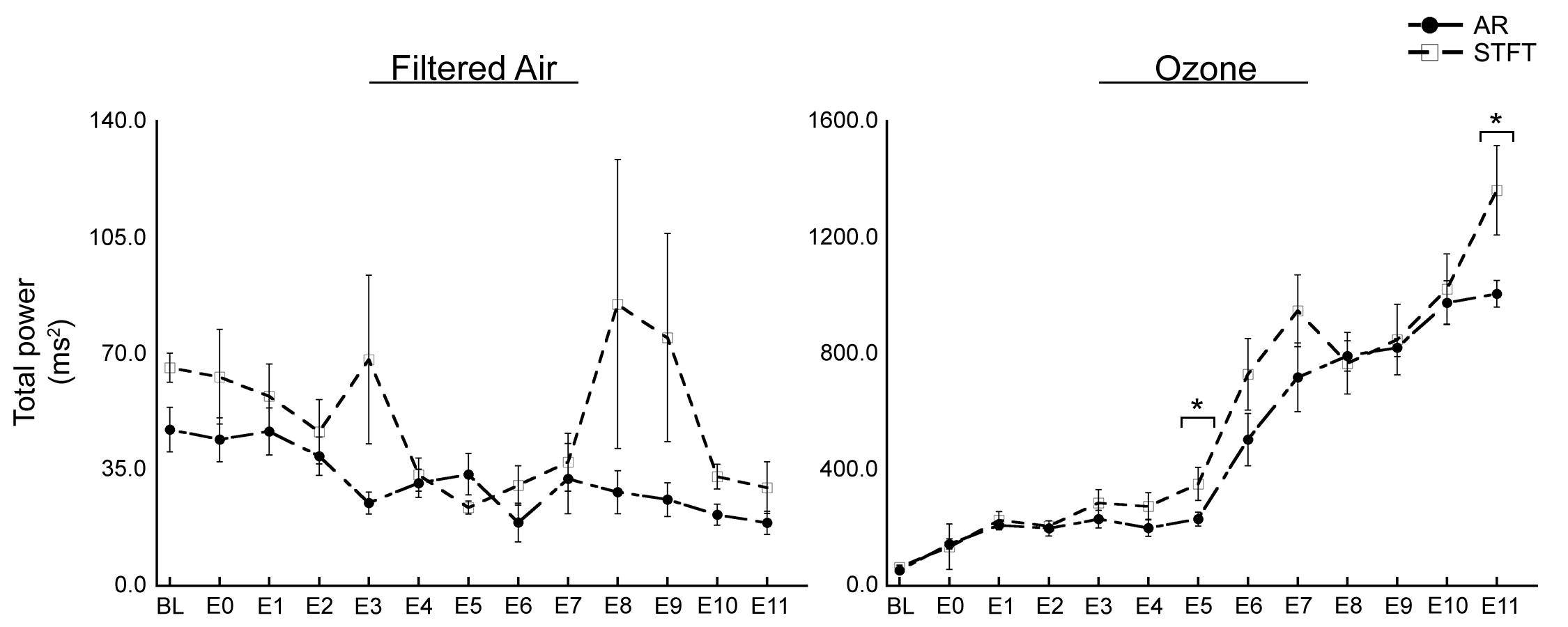

Supplement: S1 Fig — Abbreviations: O3, ozone; FA, filtered air; BL, baseline; E0, exposure start; E1-11, exposure time-points 1–11. Results are shown as the means ± SEM by exposure group. p Values of ≤ 0.05 were considered statistically significant. *Significant difference between STFT and AR results. (TIF) [file pone.0242147.s001.tif]

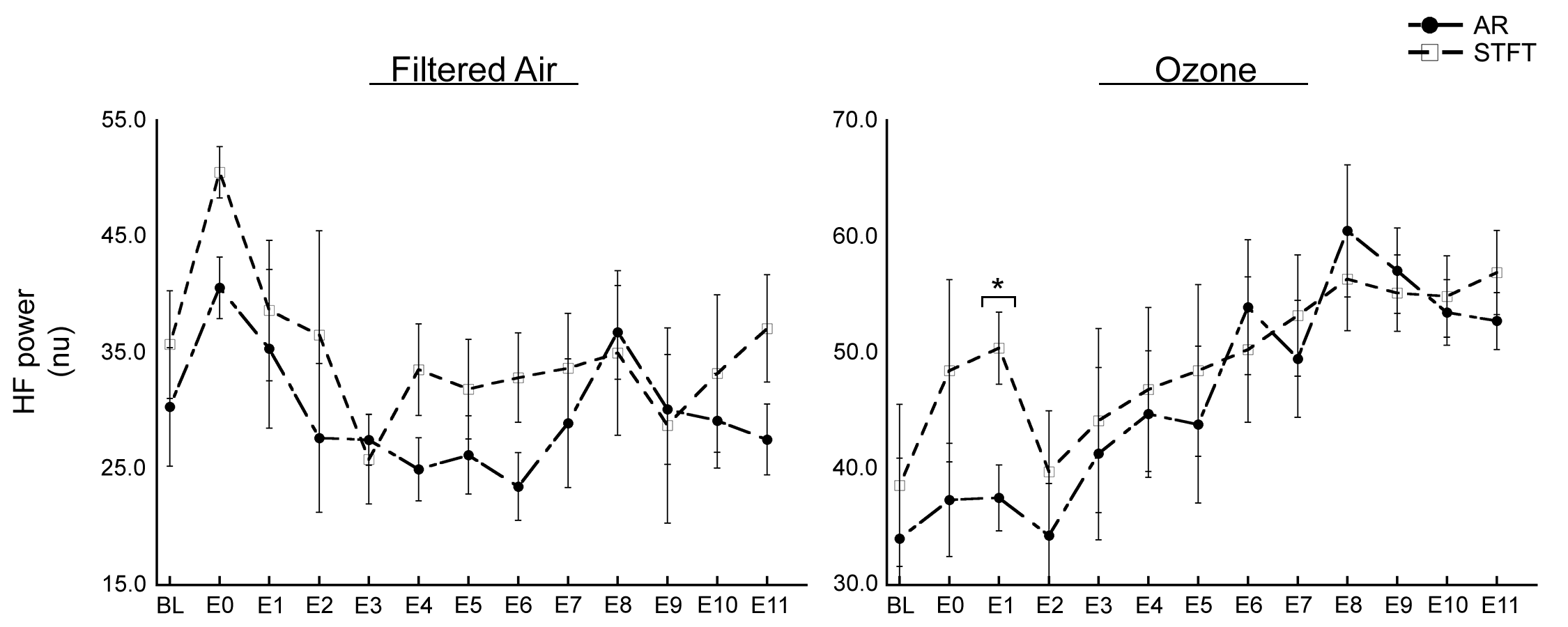

Supplement: S2 Fig — Abbreviations: O3, ozone; FA, filtered air; BL, baseline; E0, exposure start; E1-11, exposure time-points 1–11; HFn, normalized high frequency power. Results are shown as the means ± SEM by exposure group. p Values of ≤ 0.05 were considered statistically significant. *Significant difference between STFT and AR results. (TIF) [file pone.0242147.s002.tif]
