# Supplementary material for: Comparison of nonparametric and parametric methods for time-frequency heart rate variability analysis in a rodent model of cardiovascular disease
Source: PLoS One. 2020 Nov 9;15(11):e0242147. doi: 10.1371/journal.pone.0242147 (PMC7652293; doi:10.1371/journal.pone.0242147)
Supplement: S1 Table — Mean age, weight, and number of weeks post-telemeter implantation of rats on day of experimental exposure. Abbreviations: FA, filtered air; O3, ozone; wko, age in weeks; TI/E, time between telemeter implantation and experimental exposure. Values are shown as means ± SEM. (DOCX) [file pone.0242147.s003.docx]

| S1 TABLE | | |
| --- | --- | --- |
| Parameter | FA  (*n* = 5) | O_3_  (*n* = 6) |
| Age (wko) | 44.7 ± 1.4 | 44.6 ± 1.3 |
| Weight (g) | 323.4 ± 9.9 | 324.0 ± 9.6 |
| TI/E (wks) | 3.2 ± 0.8 | 3.0 ± 0.6 |
